# Supplementary material for: T-cell-derived IFN-γ suppresses T follicular helper cell differentiation and antibody responses
Source: EMBO J. 2025 Apr 1;44(9):2400–23. doi: 10.1038/s44318-025-00414-3 (PMC12048687; doi:10.1038/s44318-025-00414-3)
Supplement: Supplementary file 1 — Appendix [file 44318_2025_414_MOESM1_ESM.pdf]

Appendix for "T-cell-derived IFN-γ Suppresses T Follicular Helper Cell Differentiation and Antibody Responses"

Table of Contents:

Appendix Figure S1. *VSV and LCMV induce distinct antiviral CD4+ T cell polarization* - (page 3)

Appendix Figure S2. *$CD4^+$ T cells from the infected hosts are clustered based on the infective agent* - (page 4)

Appendix Figure S3. *Gating strategy of Tcf-1 and GzmB subsets among T-bet^+ Smarta $CD4^+$ T cells* - (page 6)

Appendix Figure S4. *Gating strategy of Tcf-1 and GzmB subsets among T-bet^+ endogeneous $CD4^+$ T cells* - (page 8)

Appendix Figure S5. *IL-12 is not involved in $CD4^+$ T cell differentiation upon LCMV infection* - (page 9)

Appendix Figure S6. *IFN-γ suppresses T follicular helper cell differentiation* - (page 11)

Appendix Figure S7. *scRNA-seq analysis of Gzmb- and Tcf7- expressing clusters* - (page 13)

Appendix Figure S8. *The IFN-γ responsible for T_{FH} suppression is produced in the first days upon infection* - (page 15)

Appendix Figure S9. *Gating strategy for immune cell subsets analyzed for production of IFN-γ* - (page 16)

Appendix Figure S10. *IFN-γ derived from group 1 ILCs is not involved in $CD4^+$ T cell polarization* - (page 17)

Appendix Figure S11. *IFN-γ derived from DCs is not involved in CD4$^+$ T cell polarization* - (page 19)

Appendix Figure S12. *IFN-γ derived from inflammatory monocytes is not involved in CD4$^+$ T cell polarization* - (page 20)

Appendix Figure S13. *CD8$^+$ T cells contribute to CD4$^+$ T cell polarization through IFN-γ and other mechanisms* - (page 22)

Appendix Figure S14. *IFN-γ from adoptively transferred CD4$^+$ T cells is sufficient for T_{FH} suppression* - (page 23)

Appendix Figure S15. *DCs sense IFN-γ upon LCMV infection* - (page 24)

Appendix Figure S16. *Exploring the role of IFN-γ across different routes of infection* - (page 26)

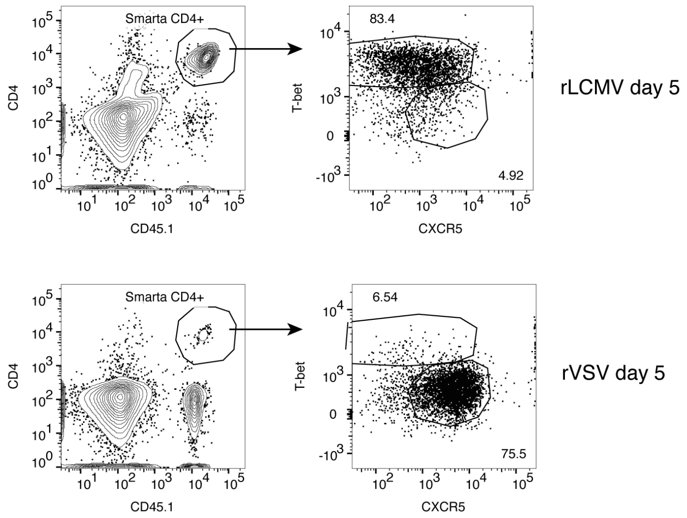

Appendix Figure S1. *VSV and LCMV induce distinct antiviral CD4+ T cell polarization.*

$0.5*10^6$ purified CD45.1$^+$ Smarta CD4+ T cells were transferred into CD45.2$^+$ WT recipients 1 day before s.c. rLCMV or rVSV infection ($1*10^5$ FFU /footpad). dLNs were analyzed 5 days post infection. Representative flow cytometry plots showing T_H1 (T-bet$^+$CXCR5$^-$) and T_{FH} (T-bet$^-$ CXCR5$^+$) cells among Smarta CD4$^+$ T cells in dLNs. Numbers represent the percentage of cells within the indicated gate.

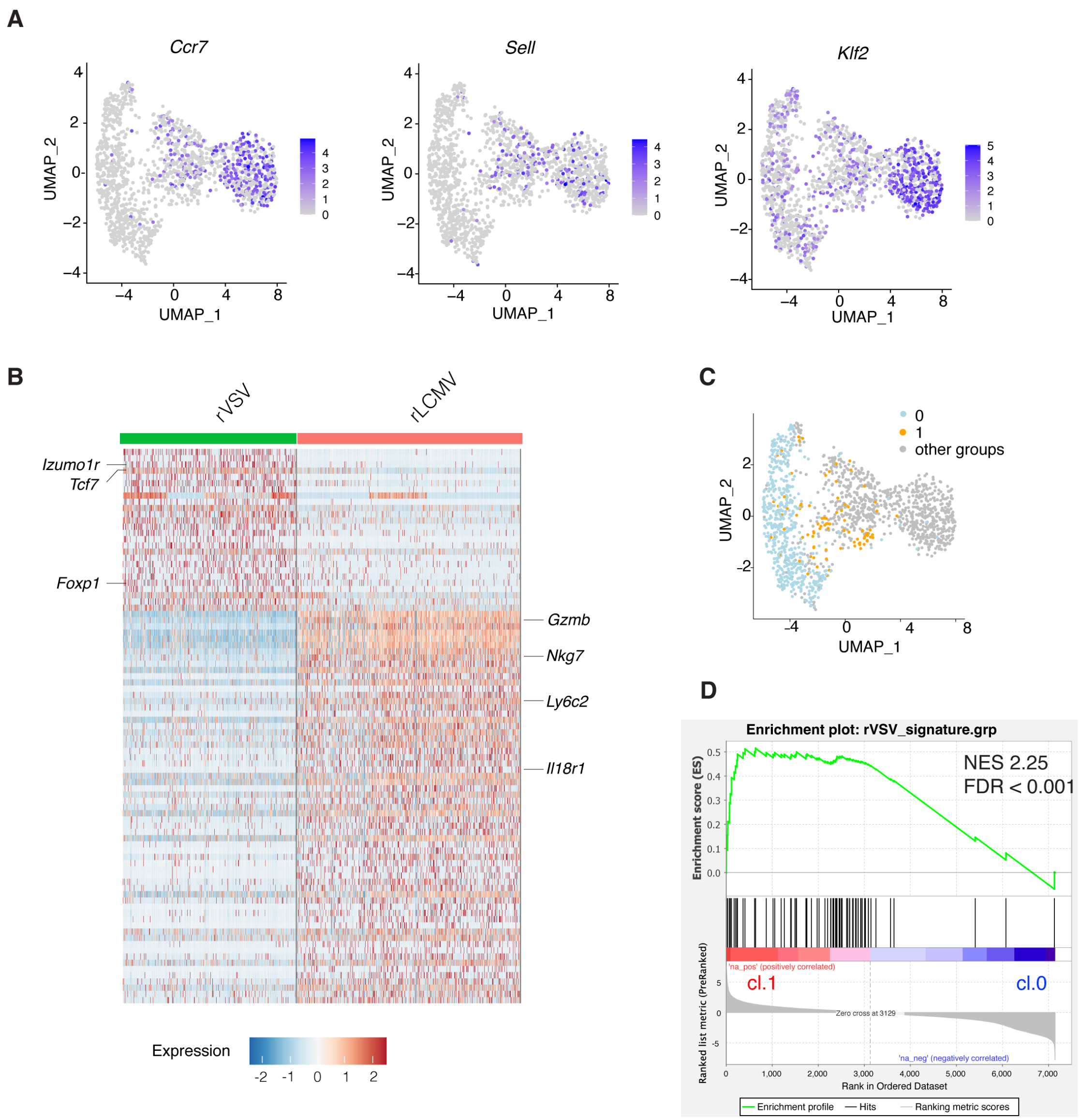

Appendix Figure S2. *CD4⁺ T cells from the infected hosts are clustered based on the infective agent.*

A) Feature plot representation of the natural-log normalized expression level of *Ccr7* (left panel), *Sell* (middle panel) and *Klf2* (right panel) on the dataset in Fig. 1B. **B)** Heatmap of normalized and scaled expression values of the 561 marker genes identifying the two groups (logFC threshold: ±0.25 and adjusted p value < 0.05 filters were applied). Color coding of the bar on the top of the

heatmap as in Fig. 1B. Noteworthy genes representative of each group are indicated. **C)** UMAP projection of dataset in Fig. 1B. Each dot corresponds to a single cell, colored according to the two unbiased clusters identified among the rLCMV cells (cluster 0 in light blue and cluster 1 in orange. Naïve and rVSV cells are colored in grey). **D)** Gene Set Enrichment Analysis (GSEA) relative to the rVSV signature enrichment in cluster 1 (Tcf7+) versus cluster 0 (Gzmb+) of Appendix Figure 1C. Genes were pre-ranked on the basis of the log2 fold change between the two clusters using the GseaPreranked Java tool (https://doi.org/10.1073/pnas.0506580102). The rVSV signature has been defined as the genes upregulated in rVSV compared to rLCMV in dataset in Appendix Figure 1B, with adj. pvalue < 0.05. NES: Normalized Enrichment Score.

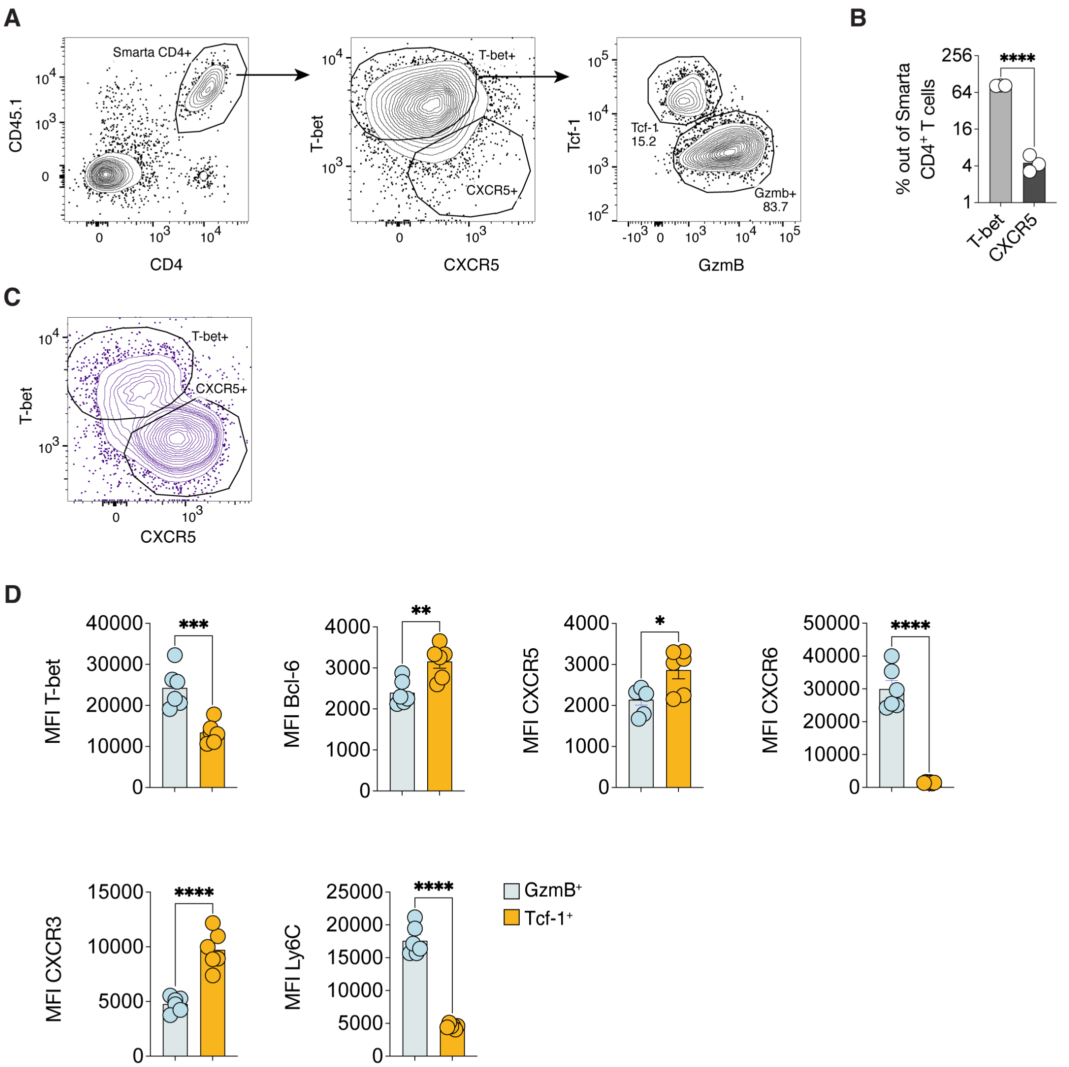

Appendix Figure S3. *Gating strategy of Tcf-1 and GzmB subsets among T-bet$^+$ Smarta CD4$^+$ T cells.*

A) $0.5*10^6$ purified CD45.1$^+$ Smarta CD4$^+$ T cells were transferred into CD45.2$^+$ WT recipients 1 day before s.c. rLCMV infection ($1*10^5$ FFU /footpad). dLNs were analyzed 5 days post infection. The gating strategy for Fig. 1F is shown. First, Smarta CD4$^+$ T were identified as CD4$^+$CD45.1$^+$ cells among all LN cells. Next, T-bet$^+$CXCR5$^-$ and T-bet$^-$CXCR5$^+$ among all Smarta CD4$^+$ T cells are shown. Finally, Tcf-1$^+$ and GzmB$^+$ cells among T-bet$^+$CXCR5$^-$ are shown. Numbers represent the percentage of cells within the indicated gate. **B)** Quantification of T-bet$^+$ and CXCR5$^+$ cells

expressed as percentages of Smarta $CD4^+$ T cells in dLNs of mice described in A. *n=3*. Mean ± SEM is shown. Data are representative of at least five independent experiments. An unpaired two-tailed t test was applied. **** *p-value* <= 0.0001. **C)** Representative plot showing T-bet^+CXCR5^- and T-bet^-CXCR5^+ cells among all cells of the LN, shown as controls for T-bet and CXCR5 staining. **D)** $0.5*10^6$ purified $CD45.1^+$ Smarta $CD4^+$ T cells were transferred into $CD45.2^+$ WT recipients 1 day before s.c. rLCMV infection ($1*10^5$ FFU /footpad). dLNs were analyzed 5 days post infection. Quantification of the MFI of T-bet, Bcl-6, CXCR5, CXCR6, CXCR3 and Ly6C on $GzmB^+$ versus Tcf-1^+ Smarta $CD4^+$ T cells in dLNs of mice described above. *n=6.* Mean ± SEM is shown. Data are representative of at least three independent experiments. An unpaired two-tailed t test was applied. * *p value* <= 0.05, ** *p value* <= 0.01, *** *p value* <= 0.001, **** *p value* <= 0.0001.

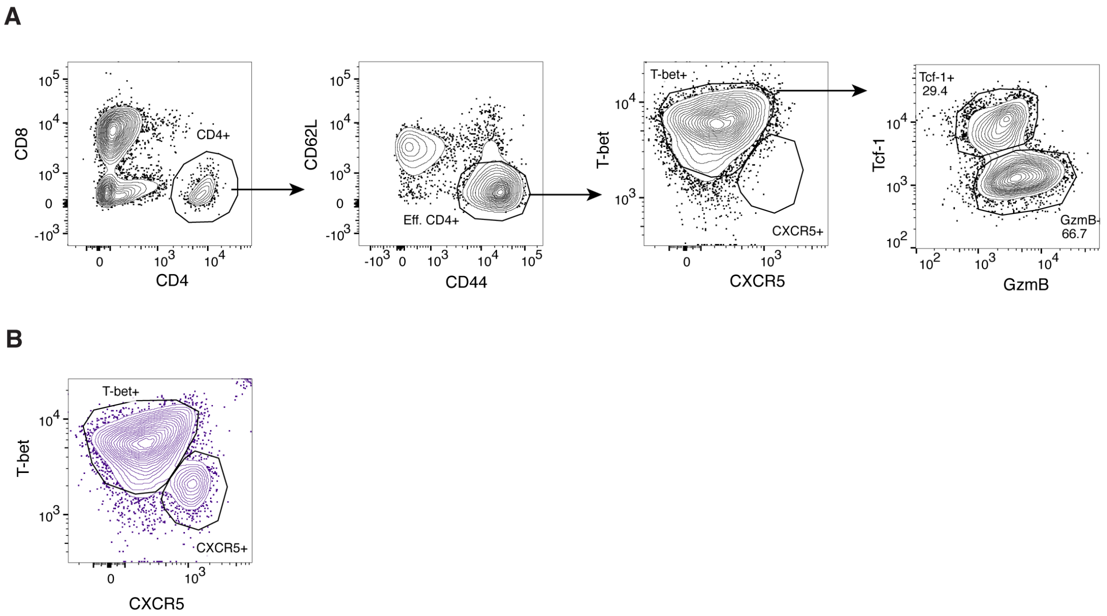

Appendix Figure S4. *Gating strategy of Tcf-1 and GzmB subsets among T-bet^{+} endogeneous CD4^{+} T cells.*

A) CD45.2^{+} WT mice were infected s.c. with rLCMV (1*10^{5} FFU /footpad) and dLNs were analyzed 7 days post infection. The gating strategy for Fig. 1H is shown. First, endogenous CD4^{+} T cells were identified as CD4^{+}CD8^{-} cells among all LN cells. Second, effector CD4^{+} T cells were identified as CD44^{+}CD62L^{-} cells among CD4^{+} T cells. Then, T-bet^{+}CXCR5^{-} and T-bet^{-}CXCR5^{+} among all effector CD4^{+} T cells are shown. Finally, Tcf-1^{+} and GzmB^{+} cells among T-bet^{+}CXCR5^{-} are shown. Numbers represent the percentage of cells within the indicated gate. **B)** Representative plot showing T-bet^{+}CXCR5^{-} and T-bet^{-}CXCR5^{+} cells among all cells of the LN, shown as controls for T-bet and CXCR5 staining.

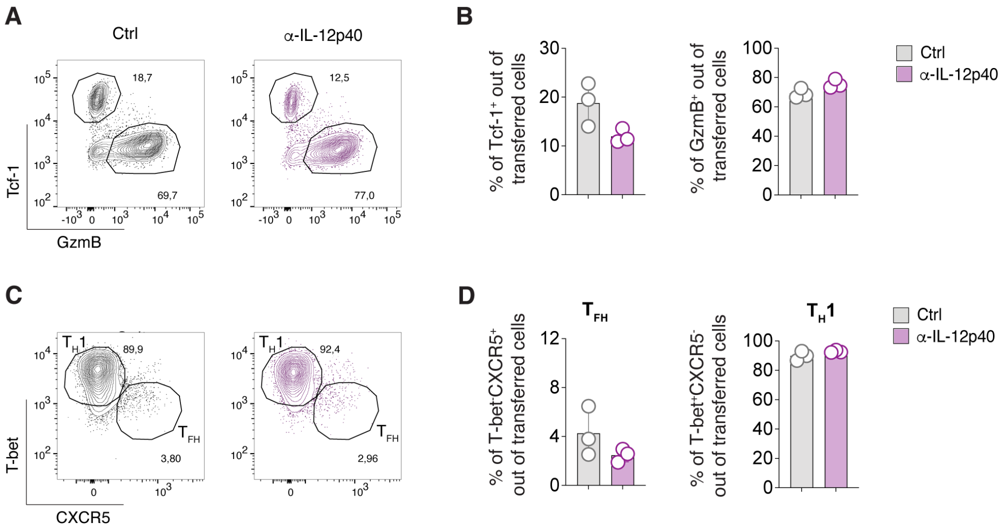

Appendix Figure S5. *IL-12 is not involved in CD4*$^+$ *T cell differentiation upon LCMV infection.* **A)** 0.5*10^6 purified CD45.1$^+$ Smarta CD4$^+$ T cells were transferred into CD45.2$^+$ WT recipients 1 day before s.c. rLCMV infection (1*10^5 FFU /footpad). CD45.2$^+$ WT recipient mice were also treated with α-IL-12p40 blocking antibody (or Isotype Ctrl) at d0 and d3 after infection. dLNs were analyzed 5 days post infection. Representative flow cytometry plot showing Tcf-1$^+$ versus GzmB$^+$ cells among Smarta CD4$^+$ T cells in dLNs. Numbers represent the percentage of cells within the indicated gate. **B**) Quantification of Tcf-1$^+$ and GzmB$^+$ cells expressed as percentages out of transferred Smarta CD4$^+$ T cells in dLNs of mice described in (A). *n=3.* Mean ± SEM is shown. Data are representative of three independent experiments. An unpaired two-tailed t test was applied. Statistics is not shown since there are no statistically significant differences between conditions. **C**) Representative flow cytometry plots showing T_H1 (T-bet$^+$CXCR5$^-$) versus T_{FH} (T-bet$^-$CXCR5$^+$) cells among Smarta CD4$^+$ T cells in dLNs of mice described in (A). Numbers represent the percentage of cells within the indicated gate. **D**) Quantification of T_{FH} and T_H1, expressed as percentages of transferred Smarta CD4$^+$ T cells, in dLNs of mice described in (A). *n=3.* Mean ± SEM is shown. Data are representative of three independent experiments. An

unpaired two-tailed t test was applied. Statistics is not shown since there are no statistically significant differences between conditions.

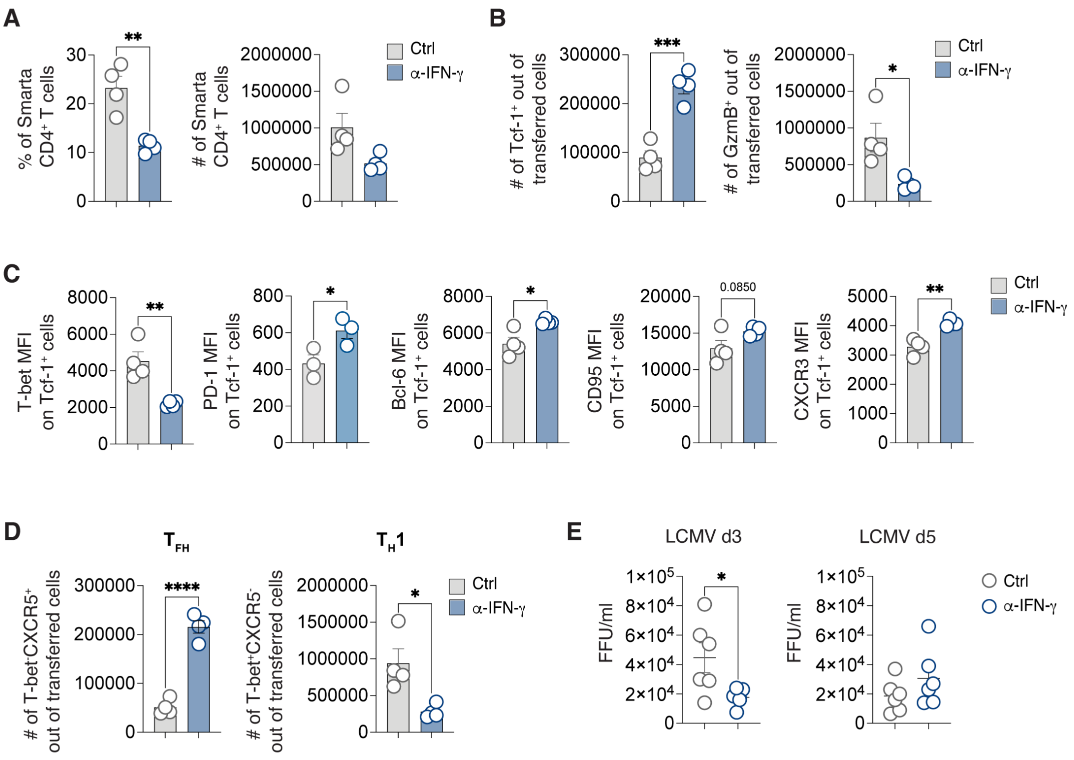

Appendix Figure S6. *IFN-γ suppresses T follicular helper cell differentiation.*

0.5*10^6 purified CD45.1$^+$ Smarta CD4+ T cells were transferred into CD45.2$^+$ WT recipients 1 day before s.c. rLCMV infection (1*10^5 FFU /footpad). CD45.2$^+$ WT recipient mice were also treated with α-IFN-γ blocking antibody (or isotype Ctrl) at day 0. dLNs were analyzed 5 days post infection. **A)** Quantification of Smarta CD4$^+$ T cells in dLNs of mice described in (A) expressed as percentages (left) and absolute numbers (right) out of total LN cells. *n=4*. Mean ± SEM is shown. Data are representative of at least three independent experiments. An unpaired two-tailed t test was applied. **p-value* <= 0.05. **B)** Quantification of Tcf-1$^+$ (left) and GzmB$^+$ (right) cells in dLNs of mice described in (A) expressed as absolute numbers out of total LN cells. *n=4*. Mean ± SEM is shown. Data are representative of at least three independent experiments. An unpaired two-tailed t test was applied. **p-value* <= 0.05, ****p-value* <= 0.001. **C)** Quantification of the MFI of T-bet, PD-1, Bcl-6, CD95, and CXCR3 on Tcf-1$^+$ Smarta CD4$^+$ T cells in dLNs of mice is

shown. *n=3,4.* Mean ± SEM is shown. Data are representative of two independent experiments. An unpaired two-tailed t test was applied. * *p value* <= 0.05, ***p-value* <= 0.01. **D)** Quantification of T_{FH} (left) and T_H1 (right), expressed as absolute numbers out of total LN cells. *n=4*. Mean ± SEM is shown. Data are representative of at least three independent experiments. An unpaired two-tailed t test was applied. **p-value* <= 0.05, *****p-value* <= 0.0001.**E)** Quantification of viral titers in dLNs at day 3 and day 5 upon LCMV infection. An unpaired two-tailed t test was applied. * *p value* <= 0.05

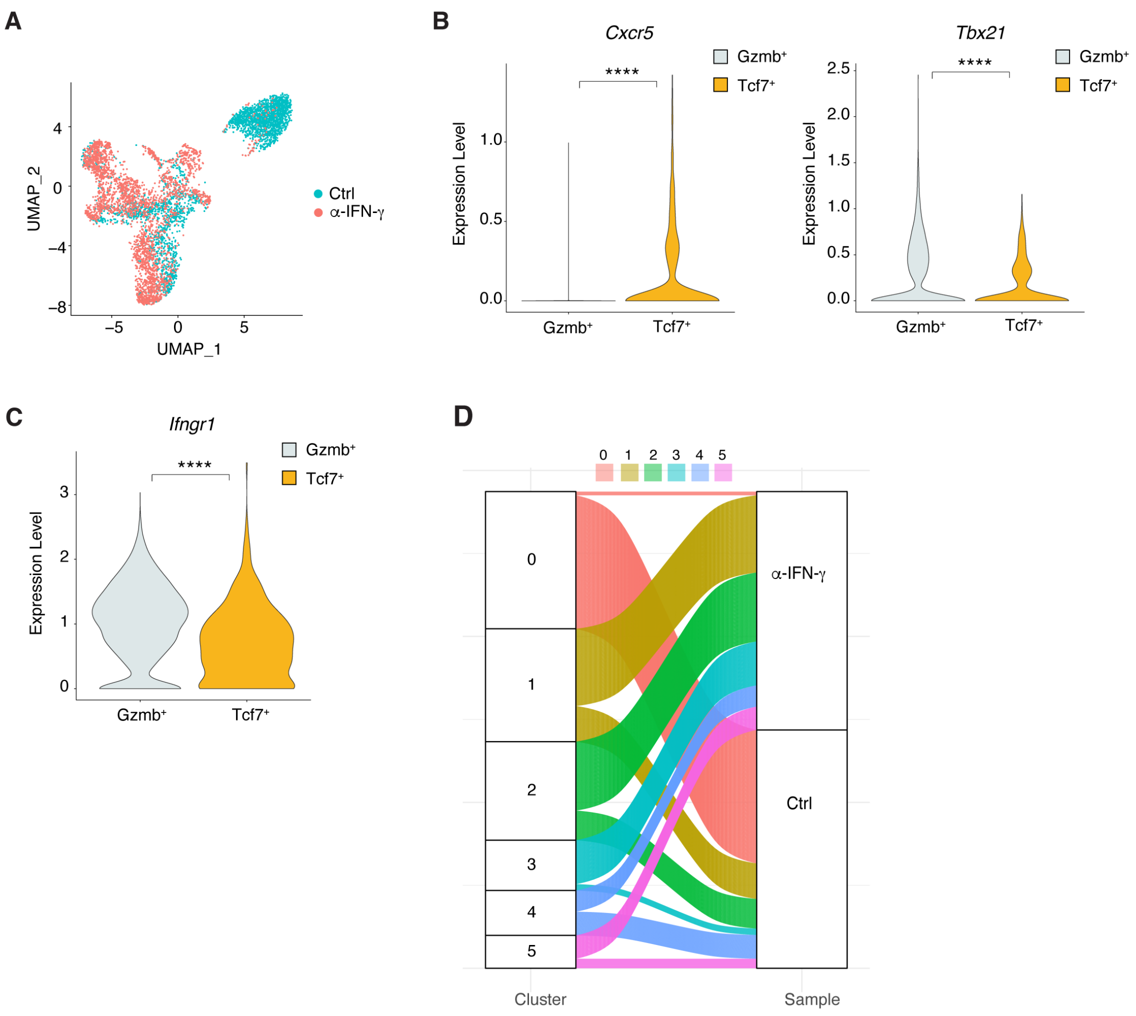

Appendix Figure S7. *scRNA-seq analysis of Gzmb- and Tcf7- expressing clusters.*

A) UMAP projection of 5,746 sorted and sequenced LCMV-specific $CD4^+$ T cells (dataset in Fig. 2). Each dot corresponds to a single cell, colored according to the condition (control cells in cyan, α-IFN-γ treated cells in red). The same number of cells for both samples has been considered (2,873 cells). **B)** Violin plot representation of the natural-log normalized expression level of *Cxcr5* (left panel) and *Tbx21* (right panel), comparing $Gzmb^+$ and $Tcf7^+$ cells belonging to dataset in A and Fig. 2. Two-tailed Mann-Whitney test has been performed, ****$p < 0.0001$. **C)** Violin plot representation of the natural-log normalized expression level of *Ifngr1*. Two-tailed Mann-Whitney

test has been performed, ****$p < 0.0001$. **D)** Alluvial plot showing the composition of unbiased clusters represented in Fig. 2G according to the condition of each cell. Cluster colors as in Fig. 2G.

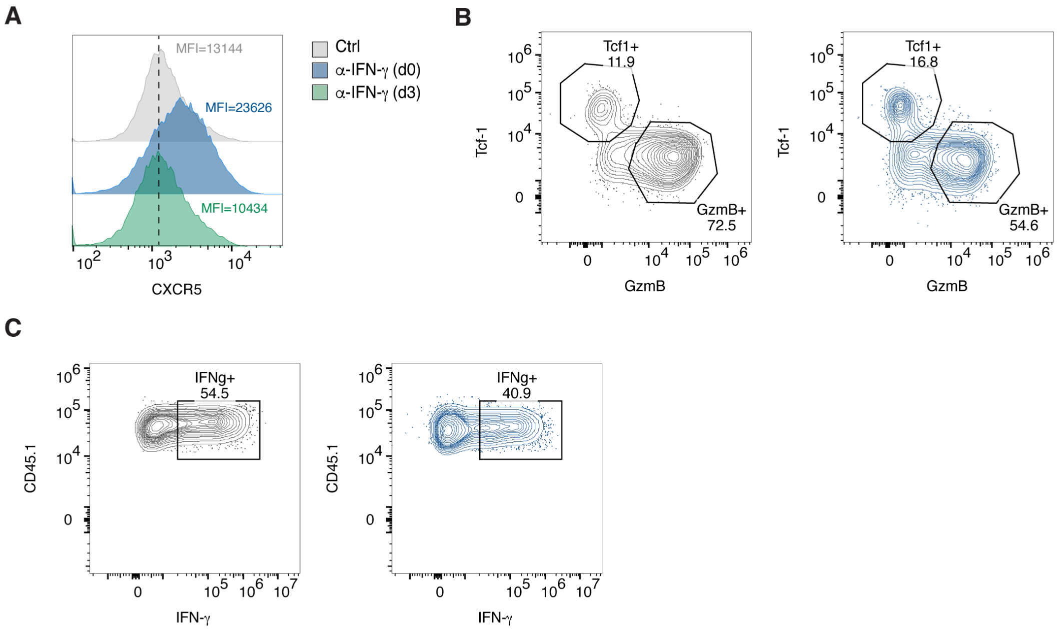

Appendix Figure S8. *The IFN-γ responsible for T_{FH} suppression is produced in the first days upon infection.*

A) 0.5*10^6 purified CD45.1$^+$ Smarta CD4$^+$ T cells were transferred into CD45.2$^+$ WT recipients 1 day before s.c. rLCMV infection (1*10^5 FFU /footpad). CD45.2$^+$ WT recipient mice were also treated with α-IFN-γ blocking antibody (or isotype Ctrl) at day 0 or d3 after infection. dLNs were analyzed 5 days post infection. Representative plots of the MFI of CXCR5 on Tcf-1$^+$ Smarta CD4$^+$ T cells are shown. **F)** 0.5*10^6 purified CD45.1$^+$ Smarta CD4$^+$ T cells were transferred into CD45.2$^+$ WT recipients 1 day before s.c. rLCMV infection (1*10^5 FFU /footpad). CD45.2$^+$ WT recipient mice were also treated with α-IFN-γ blocking antibody (or isotype Ctrl) at day 0. dLNs were analyzed 3 days post infection. Representative plots of Tcf-1$^+$ and GzmB$^+$ cells out of transferred Smarta CD4$^+$ T cells are shown. **C)** Representative plots of IFN-γ$^+$ cells out of ex-vivo restimulated Smarta CD4$^+$ T cells in dLNs of mice described in (F) are shown.

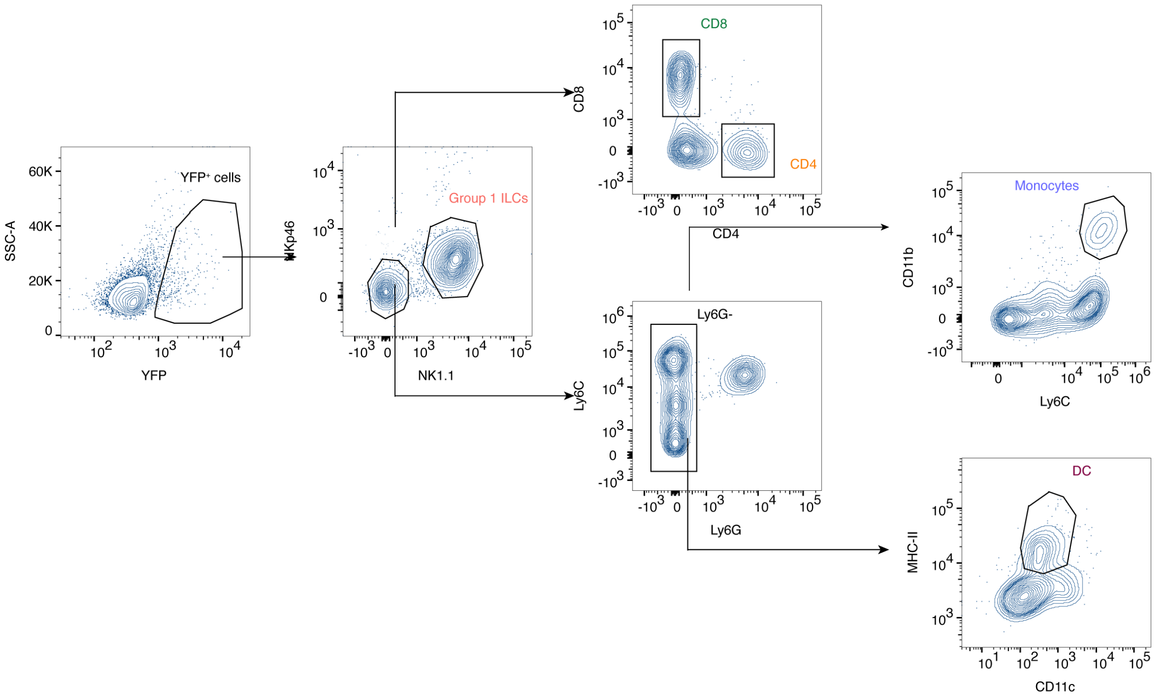

Appendix Figure S9. *Gating strategy for immune cell subsets analyzed for production of IFN-γ.* dLNs of IFN-γ-YFP mice were analyzed at 24h, 48h and 72 hours upon s.c. rLCMV infection ($1*10^5$ FFU /footpad). The gating strategy used to identify the immune cell subsets analyzed for production of IFN-γ is shown. First, group 1 ILCs were identified as NK1.1$^+$NKp46$^+$ cells out of all YFP$^+$ cells in the dLNs. Then, CD8$^+$ and CD4$^+$ T cells were identified among the NK1.1$^-$NKp46$^-$ cells. Monocytes (CD11b$^+$ Ly6C^{hi}) and DC (CD11c$^+$ MHC-II$^+$) were identified out of Ly6G$^-$ and NK1.1$^-$NKp46$^-$ cells.

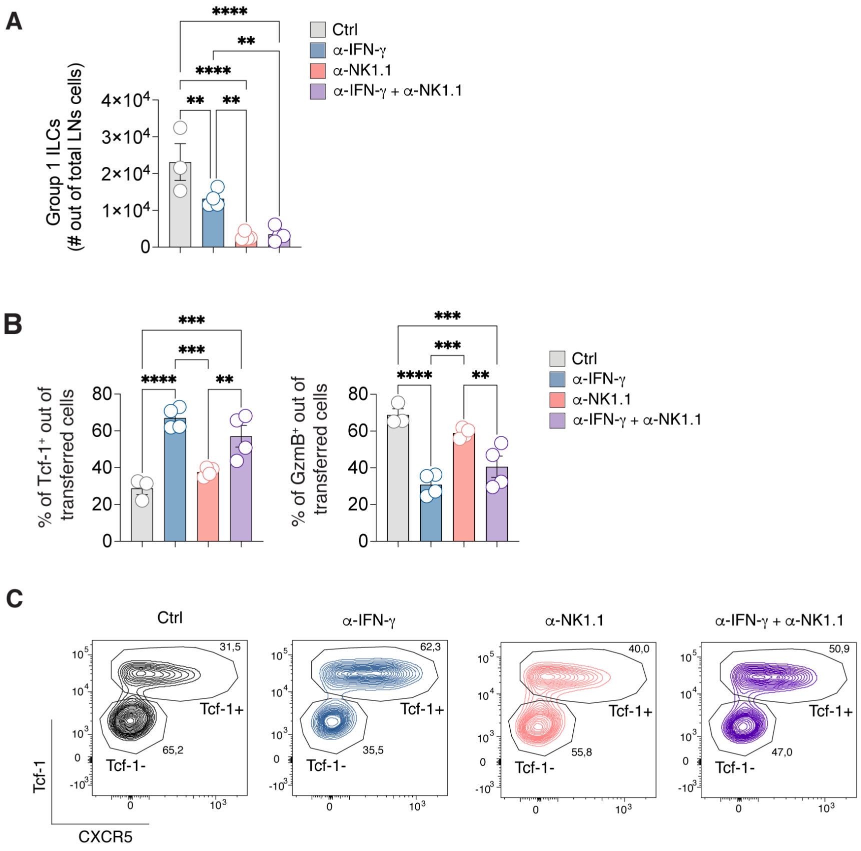

Appendix Figure S10. *IFN-γ derived from group 1 ILCs is not involved in CD4⁺ T cell polarization.*

A) $0.5*10^6$ purified $CD45.1^+$ Smarta $CD4^+$ T cells were transferred into $CD45.2^+$ WT recipients 1 day before s.c. rLCMV infection ($1*10^5$ FFU /footpad). In some conditions $CD45.2^+$ WT recipient mice were also treated with α-IFN-γ blocking antibody at day 0, α-NK1.1 antibody (d-1, d0) or both antibodies in combination. dLNs were analyzed 5 days post infection. Quantification of group 1 ILCs, expressed as absolute numbers in dLNs of described mice. *n=3* (Ctrl), 4 (α-IFN-γ, α–NK1.1, α-IFN-γ + α–NK1.1). Mean ± SEM is shown. Data are representative of three independent experiments. One-way ANOVA with uncorrected Fisher's LSD was applied. ** *p value* <= 0.01, **** *p value* <= 0.0001. **B)** Quantification of Tcf-1⁺ and GzmB⁺ cells, expressed

as percentages out of transferred Smarta $CD4^+$ T cells, in dLNs of mice described in (A). *n=3* (Ctrl), 4 (α-IFN-γ, α–NK1.1, α-IFN-γ + α–NK1.1). Mean ± SEM is shown. Data are representative of three independent experiments. One-way ANOVA with uncorrected Fisher's LSD was applied. ** *p value* <= 0.01, *** *p value* <= 0.001. **C)** Representative flow cytometry plots showing CXCR5 expression on Tcf-1^+ and Tcf-1^- Smarta $CD4^+$ T cells in dLNs of mice described in (A). Numbers represent the percentage of cells within the indicated gate.

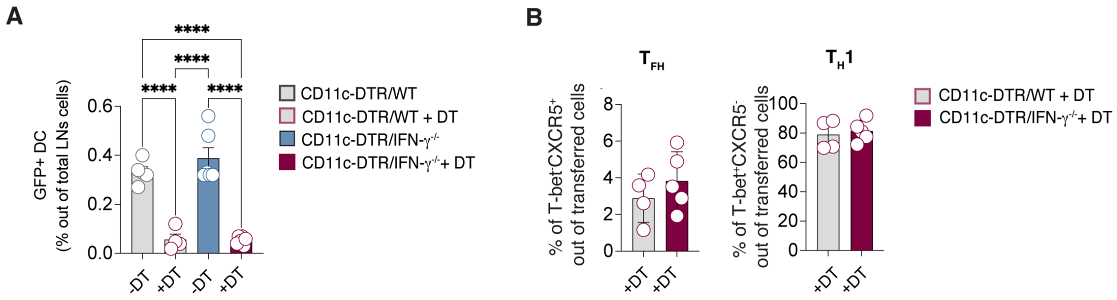

Appendix Figure S11. *IFN-γ derived from DCs is not involved in CD4$^+$ T cell polarization*

A) CD11c-DTR/WT or CD11c-DTR/IFN-γ$^{-/-}$ BM chimeras were injected with PBS or DT at day -1, 1,3 and 5 and infected s.c. with LCMV (1*10^5 FFU /footpad) at day 0. dLNs were analyzed 7 days post infection. Quantification of dendritic cells expressing GFP (CD11c$^+$MHC-II$^+$GFP$^+$) expressed as percentages out of total LNs cells is shown. *n*=4-6. Mean ± SEM is shown. Data are representative of three independent experiments. One-way ANOVA with uncorrected Fisher's LSD was applied. **** *p value* <= 0.0001. **C)** Quantification of T_{FH} and T_H1, expressed as percentages out of CD44$^+$CD62L$^-$ effector CD4$^+$ T cells, in dLNs of mice described in (A). *n*=4-6. Mean ± SEM is shown. Data are representative of three independent experiments. One-way ANOVA with uncorrected Fisher's LSD was applied. Statistics is not shown since there are no statistically significant differences between conditions.

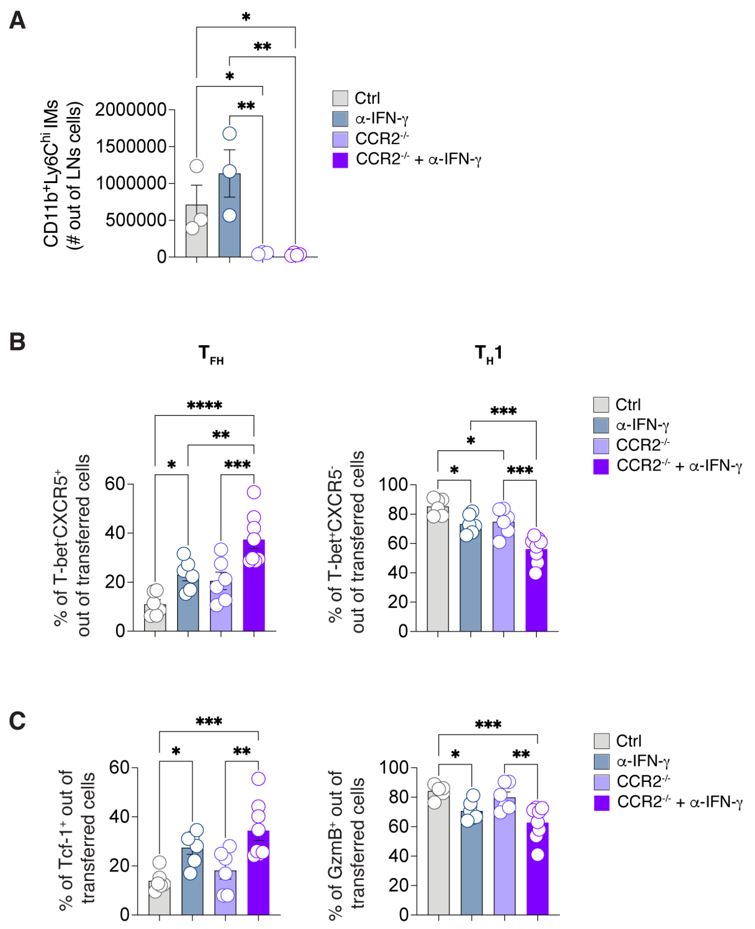

Appendix Figure S12. *IFN-γ derived from inflammatory monocytes is not involved in CD4*$^+$ *T cell polarization.*

A) 0.5*10^6 purified CD45.1$^+$ Smarta CD4$^+$ T cells were transferred into CD45.2$^+$ WT or CCR2$^{-/-}$ recipients 1 day before s.c. rLCMV infection (1*10^5 FFU /footpad). In some conditions recipient mice were also treated with α-IFN-γ blocking antibody at day 0. dLNs were analyzed 5 days post infection. Quantification of inflammatory monocytes (CD11b$^+$Ly6C$^+$), expressed as absolute numbers in dLNs of described mice. *n*=3-4. Mean ± SEM is shown. Data are representative of three independent experiments. One-way ANOVA with uncorrected Fisher's LSD was applied. * *p value* <= 0.05, ** *p value* <= 0.01. **B)** Quantification of T_{FH} and T_H1, expressed as percentages

of transferred Smarta $CD4^+$ T cells in dLNs. *n*=6-8. Mean ± SEM is shown. Data are representative of three independent experiments. One-way ANOVA with uncorrected Fisher's LSD was applied. * *p value* <= 0.05, ** *p value* <= 0.01, *** *p value* <= 0.001, **** *p value* <= 0.0001. **C)** Quantification of Tcf-1^+ and $GzmB^+$ cells, expressed as percentages out of transferred Smarta $CD4^+$ T cells in dLNs. *n*=6-8. Mean ± SEM is shown. Data are representative of three independent experiments. One-way ANOVA with uncorrected Fisher's LSD was applied. * *p value* <= 0.05, ** *p value* <= 0.01, *** *p value* <= 0.001.

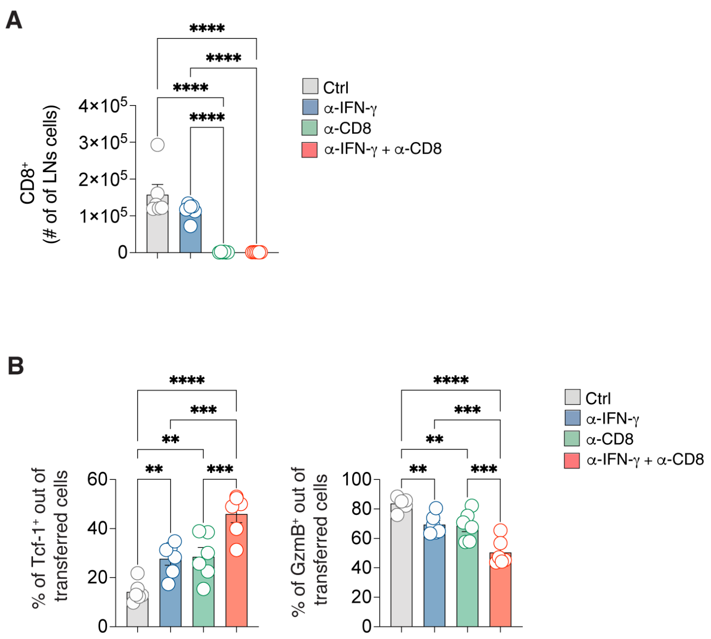

Appendix Figure S13. *CD8⁺ T cells contribute to CD4⁺ T cell polarization through IFN-γ and other mechanisms.*

A) 0.5*10^6 purified CD45.1$^+$ Smarta CD4$^+$ T cells were transferred into CD45.2$^+$ WT recipients 1 day before s.c. rLCMV infection (1*10^5 FFU /footpad). In some conditions CD45.2$^+$ WT recipient mice were also treated with α-IFN-γ blocking antibody at day 0, α-CD8 antibody (d-1, d2) or both antibodies in combination. dLNs were analyzed 5 days post infection. Quantification of CD8$^+$ T cells, expressed as absolute numbers in dLNs of described mice. *n=6*. Mean ± SEM is shown. Data are representative of two independent experiments. One-way ANOVA with uncorrected Fisher's LSD was applied. **** *p value* <= 0.0001. **B)** Quantification of Tcf-1$^+$ and GzmB$^+$ cells, expressed as percentages out of transferred Smarta CD4$^+$ T cells, in dLNs of mice described in (A). *n=6*. Mean ± SEM is shown. Data are representative of two independent experiments. One-way ANOVA with uncorrected Fisher's LSD was applied. * *p value* <= 0.05, **** *p value* <= 0.0001.

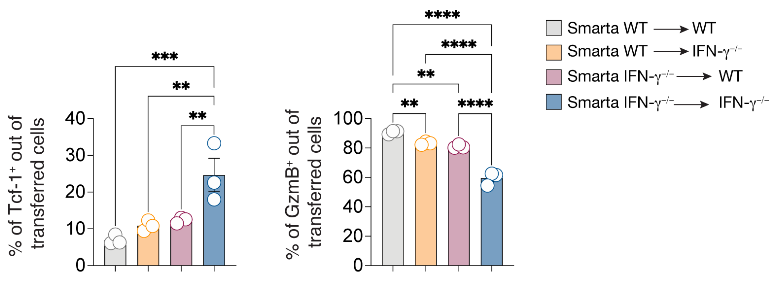

Appendix Figure S14. *IFN-γ from adoptively transferred CD4⁺ T cells is sufficient for T_{FH} suppression.*

0.5*10^6 purified $CD45.1^+$ Smarta $CD4^+$ T cells from WT or Smarta-IFN-$\gamma^{-/-}$ were transferred into $CD45.2^+$ WT or IFN-$\gamma^{-/-}$ recipients 1 day before s.c. rLCMV infection (1*10^5 FFU /footpad). dLNs were analyzed 5 days post infection. Quantification of Tcf-1^+ and $GzmB^+$ cells, expressed as percentages out of transferred Smarta $CD4^+$ T cells. *n=3*. Mean ± SEM is shown. Data are representative of three independent experiments. One-way ANOVA with uncorrected Fisher's LSD was applied. ** *p value* <= 0.01, *** *p value* <= 0.001, **** *p value* <= 0.0001.

A

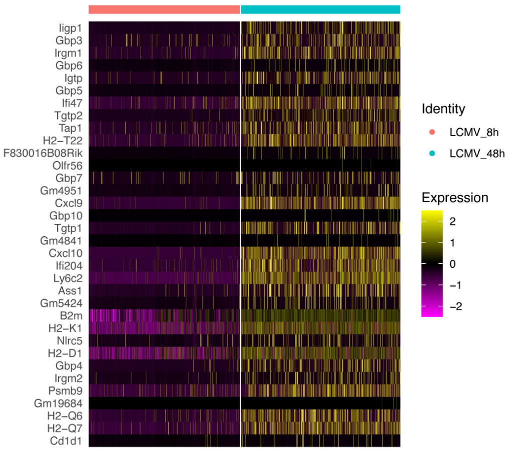

Iigp1
Gbp3
Irgm1
Gbp6
Igtp
Gbp5
Ifi47
Tgtp2
Tap1
H2−T22
F830016B08Rik
Olfr56
Gbp7
Gm4951
Cxcl9
Gbp10
Tgtp1
Gm4841
Cxcl10
Ifi204
Ly6c2
Ass1
Gm5424
B2m
H2−K1
Nlrc5
H2−D1
Gbp4
Irgm2
Psmb9
Gm19684
H2−Q6
H2−Q7
Cd1d1
Identity
LCMV_8h
LCMV_48h
Expression
2
1
0
−1
−2

B

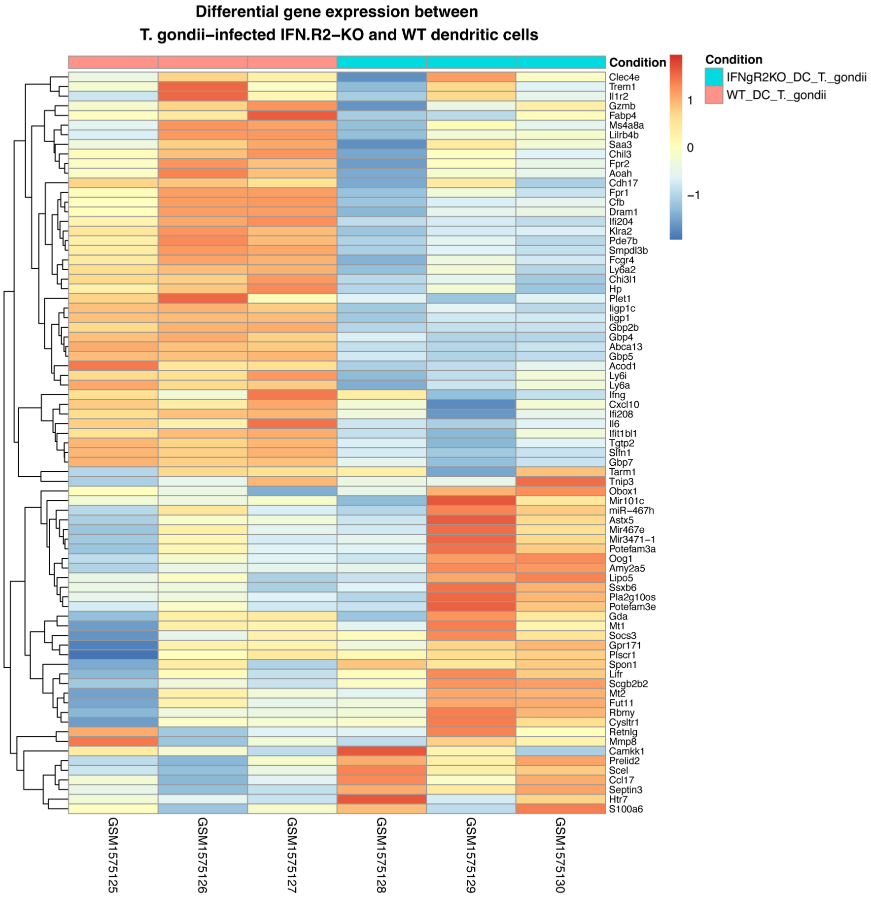

Differential gene expression between
T. gondii−infected IFN.R2−KO and WT dendritic cells
Condition
Condition
IFNgR2KO_DC_T._gondii
WT_DC_T._gondii
1
0
−1
Clec4e
Trem1
Il1r2
Gzmb
Fabp4
Ms4a8a
Lilrb4b
Saa3
Chil3
Fpr2
Aoah
Cdh17
Fpr1
Cfb
Dram1
Ifi204
Klra2
Pde7b
Smpdl3b
Fcgr4
Ly6a2
Chi3l1
Hp
Plet1
Iigp1c
Iigp1
Gbp2b
Gbp4
Abca13
Gbp5
Acod1
Ly6i
Ly6a
Ifng
Cxcl10
Ifi208
Il6
Ifit1bl1
Tgtp2
Slfn1
Gbp7
Tarm1
Tnip3
Obox1
Mir101c
miR−467h
Astx5
Mir467e
Mir3471−1
Potefam3a
Oog1
Amy2a5
Lipo5
Ssxb6
Pla2g10os
Potefam3e
Gda
Mt1
Socs3
Gpr171
Plscr1
Spon1
Lifr
Scgb2b2
Mt2
Fut11
Rbmy
Cysltr1
Retnlg
Mmp8
Camkk1
Prelid2
Scel
Ccl17
Septin3
Htr7
S100a6
GSM1575125
GSM1575126
GSM1575127
GSM1575128
GSM1575129
GSM1575130

Appendix Figure S15. *DCs sense IFN-γ upon LCMV infection.*

A) Heatmap of normalized and scaled expression values of the marker genes identifying the Ifng signature as reported in {Singhania et al., 2019 #10618}. DCs sorted from mice infected with LCMV for 8 or 48 hours (published dataset in {De Giovanni et al., 2020 #29499}) were compared.

B) Heatmap visualization of differentially expressed genes comparing T. Gondii-infected conditions, with three biological replicates per condition shown in columns. The analysis includes the top 50 upregulated genes from both IFNyR2-KO infected vs. control and WT infected vs. control comparisons, showing their expression patterns in T. gondii-infected IFNyR2-KO versus T. gondii-infected wild-type samples. Expression values are represented as z-scores, where red indicates upregulation (1) and blue indicates downregulation (-1).

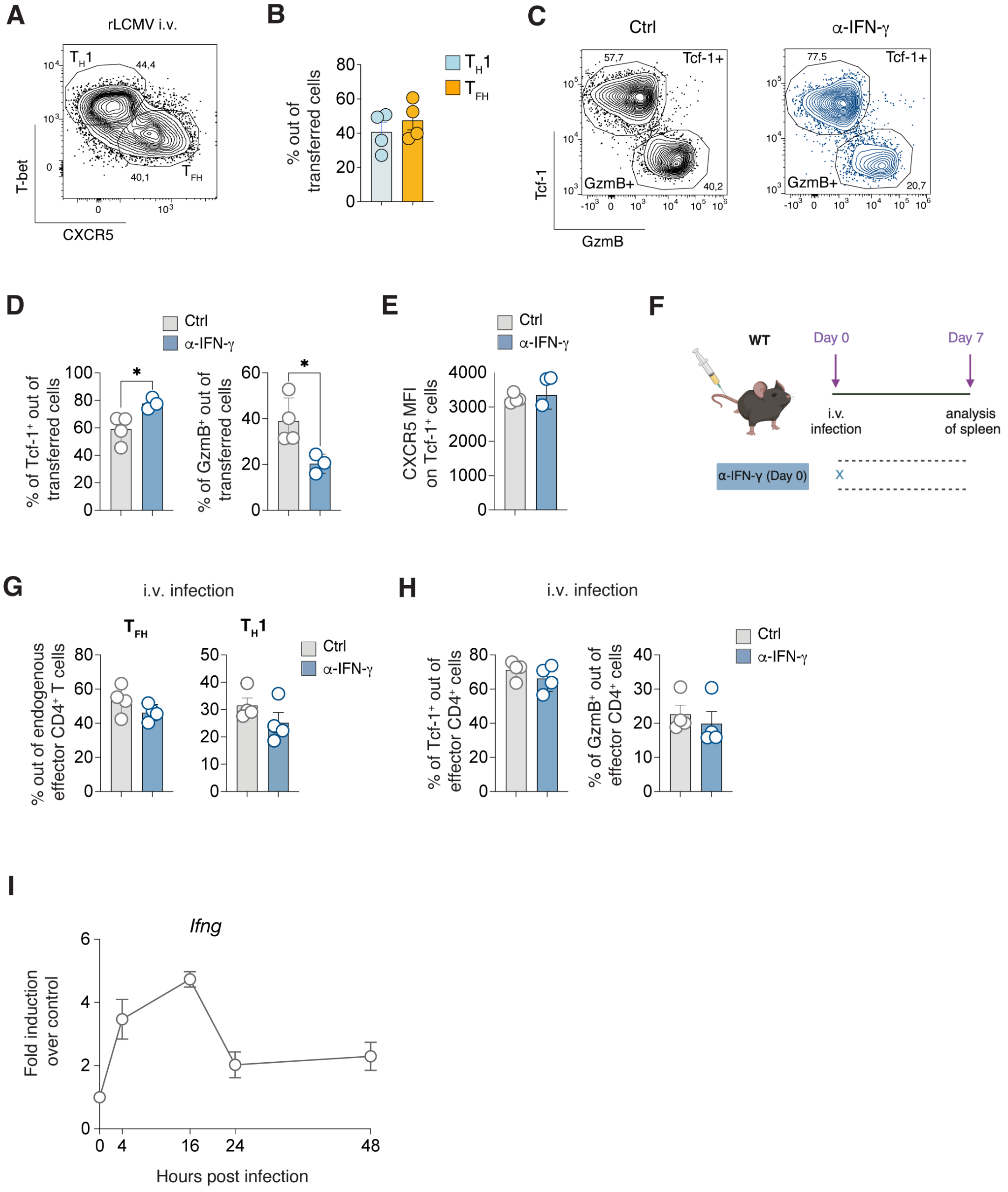

Appendix Figure S16. *Exploring the role of IFN-γ across different routes of infection.*

A) 0.5*10^6 purified CD45.1$^+$ Smarta CD4+ T cells were transferred into CD45.2$^+$ WT recipients 1 day before intravenous (i.v.) rLCMV infection (2*10^5 FFU). CD45.2$^+$ WT recipient mice were also treated with α-IFN-γ blocking antibody (or isotype Ctrl) at day 0. Spleens were analyzed 5 days post infection. Representative flow cytometry plots showing T_H1 (T-bet$^+$CXCR5$^-$) and T_{FH}

(T-bet$^-$CXCR5$^+$) cells among Smarta CD4$^+$ T cells in dLNs. Numbers represent the percentage of cells within the indicated gate. **B)** Quantification of T_{FH} and T_H1, expressed as percentages out of transferred Smarta CD4$^+$ T cells in spleens of mice described in (A). *n*=4. Mean ± SEM is shown. Data are representative of three independent experiments. An unpaired two-tailed t test was applied. Statistics is not shown since there are no statistically significant differences between conditions. **C)** Representative flow cytometry plots showing Tcf-1$^+$ versus GzmB$^+$ cells among Smarta CD4$^+$ T cells in spleens. Numbers represent the percentage of cells within the indicated gate. **D)** Quantification of Tcf-1$^+$ and GzmB$^+$ cells, expressed as percentages out of transferred Smarta CD4$^+$ T cells in spleens of mice described in (A). *n*=4 (Ctrl), 3 (α-IFN-γ). Mean ± SEM is shown. Data are representative of three independent experiments. An unpaired two-tailed t test was applied. * *p value* <= 0.05. **E)** Quantification of the MFI of CXCR5 on Tcf-1$^+$ Smarta CD4$^+$ T cells in spleens of mice described in (A). *n*=4 (Ctrl), 3 (α-IFN-γ). Mean ± SEM is shown. Data are representative of three independent experiments. An unpaired two-tailed t test was applied. Statistics is not shown since there are no statistically significant differences between conditions. **F)** WT mice were infected i.v. ($2*10^5$ FFU) with rLCMV and spleens were analyzed 7 days upon infection. WT mice were also treated with α-IFN-γ blocking antibody (or isotype Ctrl) at day 0. **G)** Quantification of T_{FH} (left) and T_H1 (right), expressed as percentages out of endogenous effector CD4$^+$ T cells in spleens of i.v. infected mice described in (E). *n*=4. Mean ± SEM is shown. Data are representative of three independent experiments. An unpaired two-tailed t test was applied but no statistically significant differences were detected. **H)** Quantification of Tcf-1$^+$ and GzmB$^+$ cells, expressed as percentages out of endogenous effector CD4$^+$ T cells in spleens of i.v. infected mice. *n*=4. Mean ± SEM is shown. Data are representative of three independent experiments. An unpaired two-tailed t test was applied but no statistically significant differences were detected. **I)**

WT mice were immunized s.c. with MPLA+ RBD-S1. Analysis of *Ifng* gene expression at 0, 4, 8, 16, 24 and 48 hours in dLN of immunized mice is shown. *n=6.* Mean ± SEM is shown.
