# Supplementary figures and images for: T-cell-derived IFN-γ suppresses T follicular helper cell differentiation and antibody responses
Source: EMBO J. 2025 Apr 1;44(9):2400–23. doi: 10.1038/s44318-025-00414-3 (PMC12048687; doi:10.1038/s44318-025-00414-3)

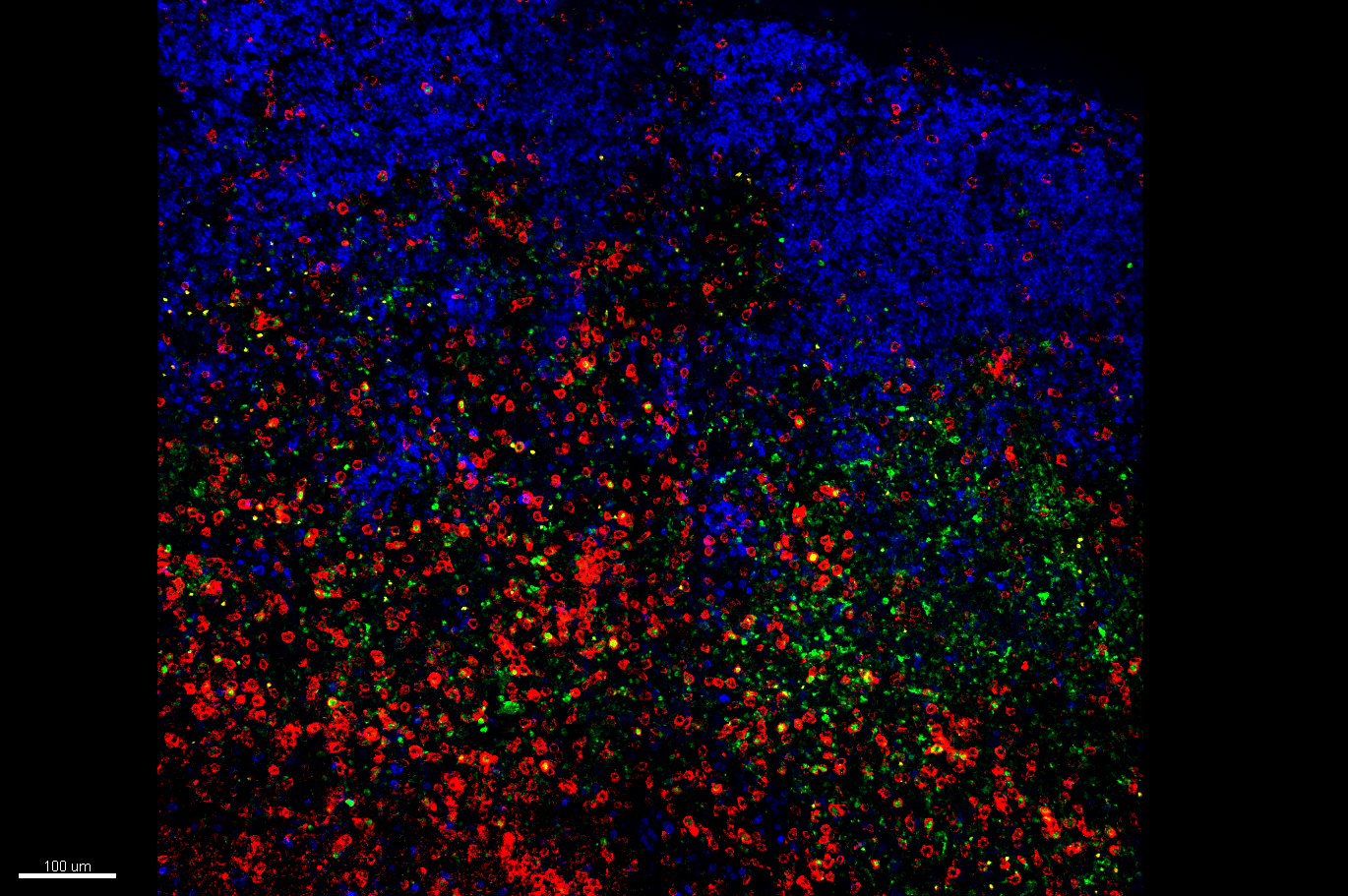

Supplement: Supplementary file 8 — Source data Fig. 3 [file 44318_2025_414_MOESM8_ESM.zip › Figure 3/3A/MM#8 5A PBS_m_TileScan 001 TileScan_001_Merging yes cropped 1_2023-11-14T15-25-11.704.tif]

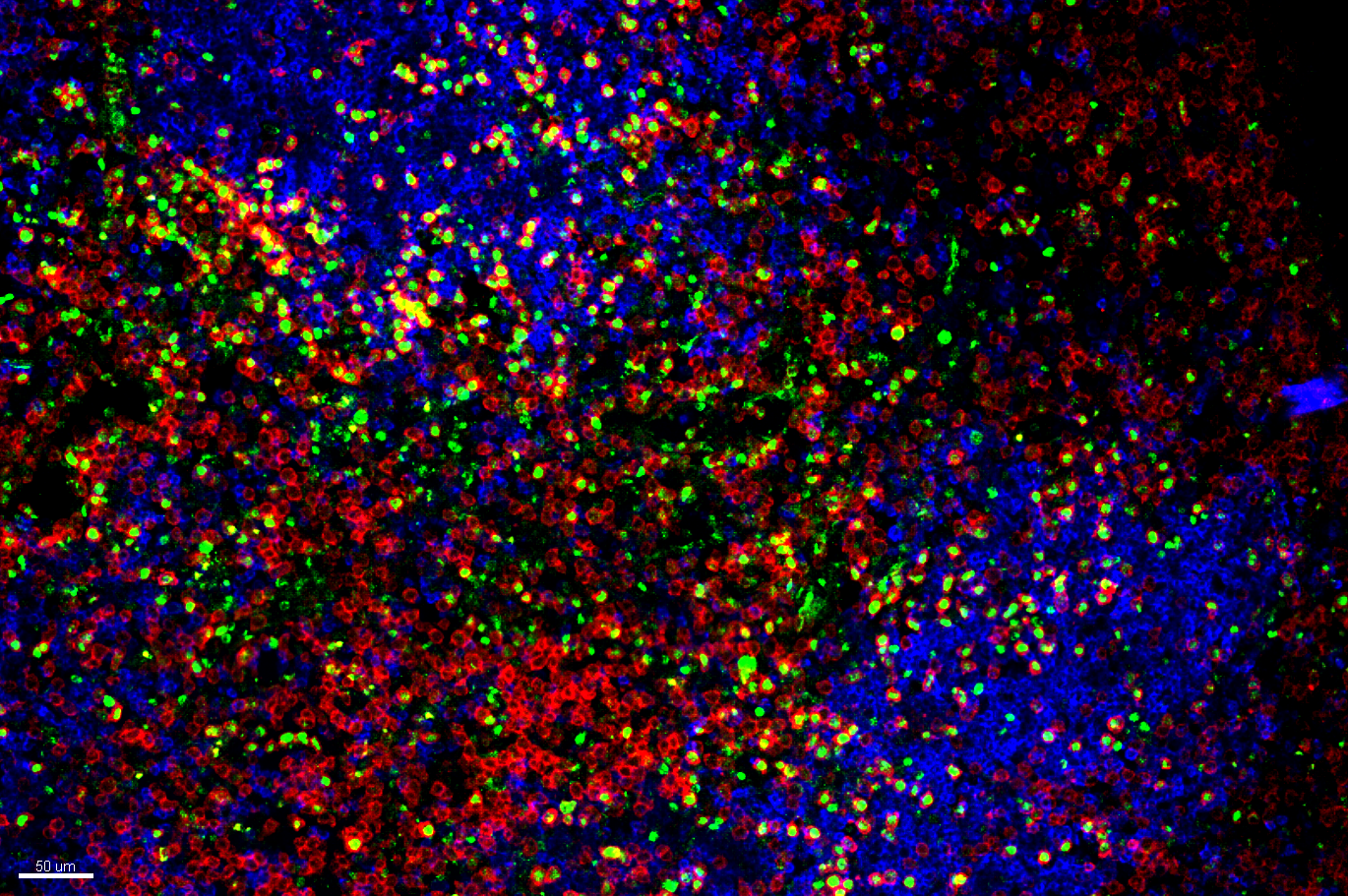

Supplement: Supplementary file 8 — Source data Fig. 3 [file 44318_2025_414_MOESM8_ESM.zip › Figure 3/3A/MM#8 9A aIFNg_q_TileScan 001 TileScan_001_Merging yes cropped_2023-11-14T15-24-03.043.tif]

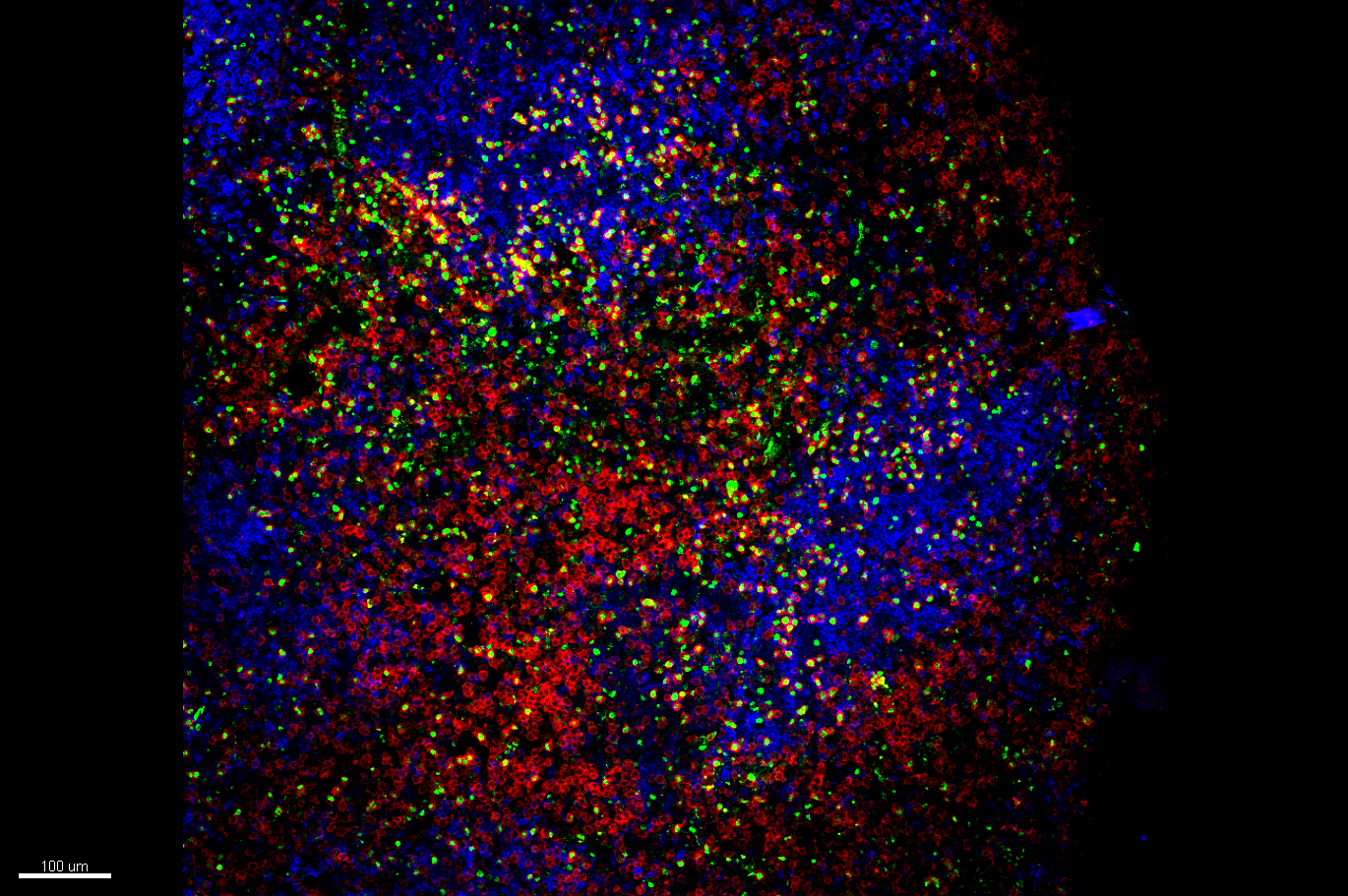

Supplement: Supplementary file 8 — Source data Fig. 3 [file 44318_2025_414_MOESM8_ESM.zip › Figure 3/3A/MM#8 9A aIFNg_q_TileScan 001 TileScan_001_Merging yes cropped_2023-11-14T15-24-14.473.tif]

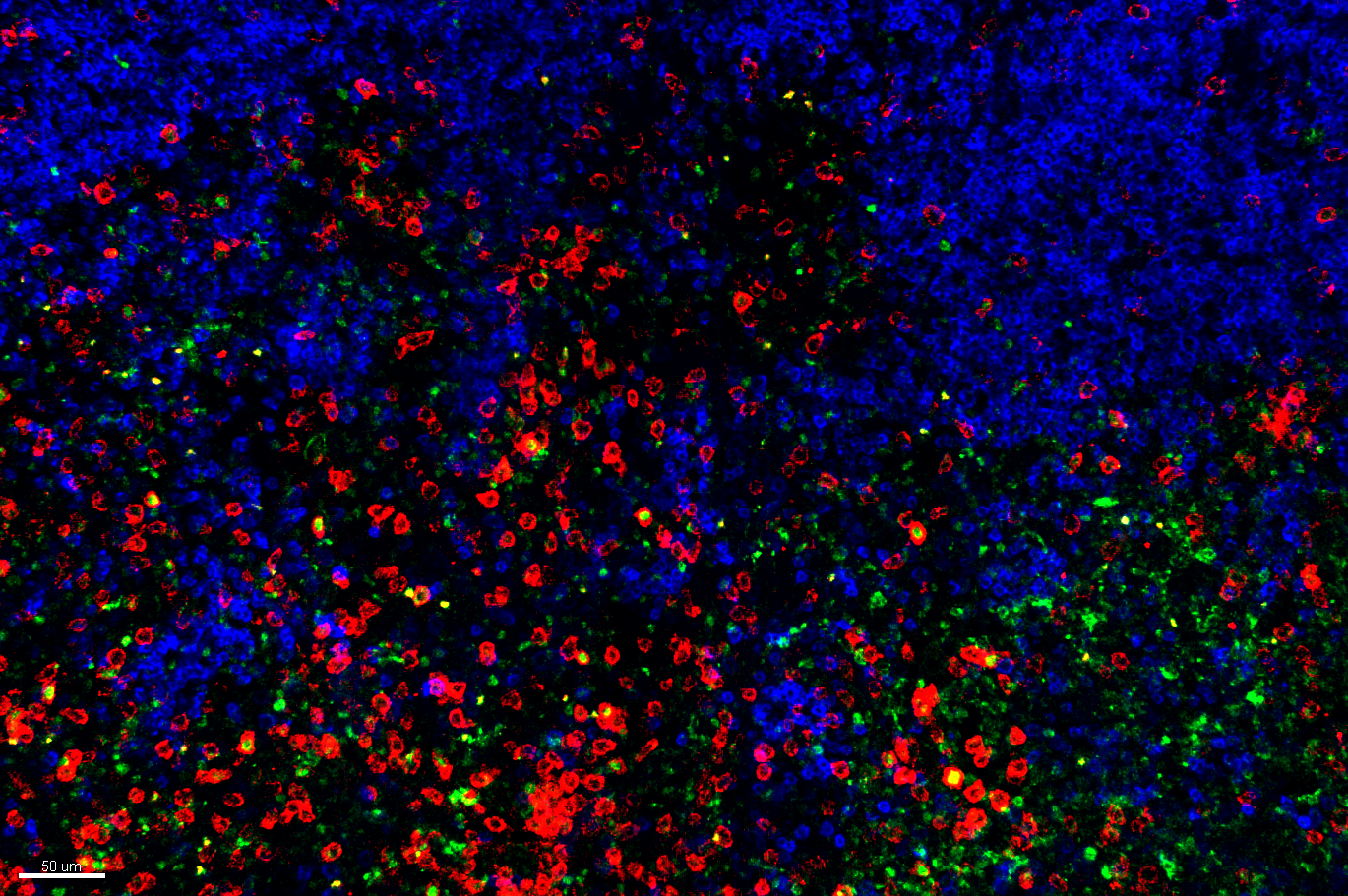

Supplement: Supplementary file 8 — Source data Fig. 3 [file 44318_2025_414_MOESM8_ESM.zip › Figure 3/3A/MM#8 5A PBS_m_TileScan 001 TileScan_001_Merging yes cropped 1_2023-11-14T15-26-06.116.tif]
